# PdMo Bimetallene as a High-Performance Electrochemical Sensor for the Selective Detection of Dopamine

**DOI:** 10.3390/ijms27093861

**Published:** 2026-04-27

**Authors:** Yuting Zhong, Lei Li, Yunbing Wang

**Affiliations:** National Engineering Research Center for Biomaterials, College of Biomedical Engineering, Sichuan University, Chengdu 610065, China; zhongyuting1@stu.scu.edu.cn (Y.Z.); leili_666@stu.scu.edu.cn (L.L.)

**Keywords:** dopamine, PdMo bimetallene, electrochemical biosensor

## Abstract

Dopamine (DA) is a crucial catecholamine neurotransmitter, and its abnormal levels are closely associated with neurological disorders such as Parkinson’s disease. Electrochemical sensing technology offers a rapid and cost-effective platform for DA detection; however, it often suffers from interference from coexisting biomolecules such as ascorbic acid (AA) and uric acid (UA). In this study, we report a novel electrochemical biosensor based on PdMo bimetallene, a nanomaterial synthesized via a facile wet-chemical approach, aiming to enhance the detection performance and selectivity for DA. PdMo bimetallene is a highly curved, atomically thin two-dimensional nanosheet featuring abundant strained sites and a high density of active centers, enabling the selective and sensitive detection of DA. The results demonstrate that the as-prepared PdMo bimetallene-modified glassy carbon electrode (GCE) exhibits excellent electrocatalytic activity toward the oxidation of DA. The sensor displays a good linear response over the concentration range from 10 nM to 200 µM, with an ultrahigh sensitivity of 80 µA·µM^−1^ cm^−2^ and a low detection limit of 0.14 µM (S/N = 3). Owing to the synergistic electronic effect between Pd and Mo, the high density of exposed active sites, and the unique strained lattice structure of the bimetallene, the sensor enables accurate determination of DA concentrations even in the presence of interfering species such as AA and UA. In summary, the successfully fabricated PdMo bimetallene-based sensor offers the advantages of low cost, facile synthesis, a wide linear range, and high sensitivity, positioning it as a promising candidate for neurotransmitter detection applications.

## 1. Introduction

Dopamine (3,4-dihydroxyphenethylamine, DA), as one of the most representative catecholamine neurotransmitters in the central nervous system, is extensively involved in the regulation of multiple physiological processes, including motor coordination, emotional regulation, cognitive processing, reward feedback, and endocrine homeostasis [1,2]. In a healthy organism, DA levels in bodily fluids and brain tissues are typically maintained at low concentrations, with its dynamic balance being essential for normal neurological function [3]. Disruption of this balance, leading to abnormal fluctuations in DA levels, often serves as a pathological hallmark of various neuropsychiatric disorders, such as Parkinson’s disease, schizophrenia, attention deficit hyperactivity disorder, and depression [4]. Therefore, the development of rapid, accurate, and cost-effective analytical methods for DA detection is of great significance not only for the early diagnosis and therapeutic evaluation of these diseases but also for providing critical technical tools for fundamental research in neurobiology.

Among various detection techniques, electrochemical analysis methods have garnered considerable attention due to their unique advantages [5,6]. Compared with traditional techniques such as high-performance liquid chromatography (HPLC) [7,8], spectrophotometry [9], chemiluminescence [10], and capillary electrophoresis [11], electrochemical sensors offer high sensitivity, fast response, operational simplicity, low equipment cost, and ease of miniaturization and online monitoring [12]. With the rapid advancement of wearable devices and portable analytical instruments, electrochemical sensors hold significant promise for applications in home healthcare monitoring and point-of-care testing. However, a long-standing technical challenge in the electrochemical detection of DA in complex biological samples (e.g., serum, urine, or cerebrospinal fluid) is interference from coexisting electroactive species [13]. Among these, ascorbic acid (AA) and uric acid (UA) are the most common interferents, with oxidation potentials that strongly overlap with that of DA [14,15]. At bare, unmodified electrode surfaces (such as glassy carbon (GCE) or gold electrodes), the oxidation signals of these three species often merge into a single, broad, and poorly resolved peak, severely compromising detection accuracy and selectivity. Consequently, the design of novel electrode materials capable of selectively catalyzing the oxidation of DA while effectively suppressing responses from interferents has emerged as a central challenge in this field.

The emergence of two-dimensional nanomaterials has provided new opportunities for enhancing the performance of electrochemical sensing interfaces [16,17]. Typical two-dimensional materials such as graphene, MXene, and transition metal dichalcogenides have been widely employed in the construction of high-performance electrochemical sensors owing to their atomic thickness, ultrahigh specific surface area, excellent electrical conductivity, and abundant surface active sites [18]. Such materials not only significantly accelerate electron transfer processes at the electrode interface but also enable specific recognition of target molecules through surface functionalization strategies. In recent years, a novel class of two-dimensional materials known as metallenes has rapidly emerged as a frontier research focus in the fields of electrochemistry and materials science. Metallenes are atomically thin two-dimensional nanomaterials composed of coordinatively unsaturated metal atoms, exhibiting exciting characteristics that integrate atomic economy, ultrathin structures, quantum confinement effects, photonic properties, and catalytic activity, rendering them highly promising for a wide range of electrocatalytic applications [19,20,21]. Furthermore, bimetallenes also exhibit synergistic effects between the two metallic components, potentially delivering superior performance beyond that of conventional nanomaterials in areas such as electrocatalysis and energy conversion. Common bimetallene systems include PdMo, PdRu, and PdIr, which typically possess abundant surface defects, unique strained lattice structures, and strong electronic coupling effects [22,23,24]. Studies have shown that lattice strain can effectively modulate the d-band center position of bimetallenes, thereby optimizing the adsorption/desorption behavior of reaction intermediates on the catalyst surface and significantly enhancing catalytic performance [23,25]. This mechanism of performance enhancement achieved through strain engineering and electronic structure regulation offers new insights for the design of electrochemical sensors.

Palladium-based nanomaterials have been extensively studied due to their excellent electrocatalytic oxidation capability toward small organic molecules such as DA, glucose, and alcohols [26]. However, pure palladium (Pd) materials are susceptible to poisoning by the adsorption of reaction intermediates during catalysis, leading to a decay in catalytic activity over time. Alloying with early transition metals such as molybdenum (Mo) not only effectively modulates the electronic structure of Pd but also significantly enhances its catalytic stability. Specifically, the incorporation of Mo atoms induces a downward shift in the d-band center of Pd, thereby optimizing the adsorption and desorption behavior of reactants and intermediates on the catalyst surface [22,27,28]. This mechanism has been systematically demonstrated in the oxygen reduction reaction and in the oxidation of various small organic molecules. Although bimetallenes have shown great potential in the field of electrocatalysis, their application in electrochemical sensing, particularly for the detection of neurotransmitters, remains in its infancy, with only limited reports available. Combining the unique structural advantages of bimetallenes with the technical requirements of electrochemical sensing holds promise for opening new avenues toward the development of highly sensitive and selective biosensors.

Based on the above background, in this study, PdMo bimetallene was employed as an electrode modification material to construct an electrochemical sensing platform for the selective detection of DA. We propose the following scientific hypothesis: the unique two-dimensional structure, high density of exposed active sites, electronic synergy between Pd and Mo, and strain-induced surface chemistry of PdMo bimetallene will synergistically enhance the electrocatalytic oxidation kinetics of DA while achieving high selectivity by modulating the reaction energy barriers of AA and UA at the electrode interface. To validate this hypothesis, this study systematically conducted the controllable synthesis, morphological and structural characterization, optimization of electrode interface modification, and electrochemical performance evaluation of PdMo bimetallene. Key analytical performance metrics of the sensor for DA detection, including linear response range, sensitivity, detection limit, anti-interference capability, reproducibility, and repeatability, were comprehensively investigated. Experimental results demonstrate that the PdMo bimetallene-modified electrode enables highly sensitive and selective detection of DA over a wide concentration range, with overall performance significantly outperforming most currently reported sensors based on two-dimensional materials and noble metal nanoparticles. This study not only provides new design strategies for the development of high-performance electrochemical sensing materials but also expands the application boundaries of bimetallenes in biomedical analysis.

## 2. Results

### 2.1. Morphological and Structural Characterization of PdMo Bimetallene

**Figure 1:**
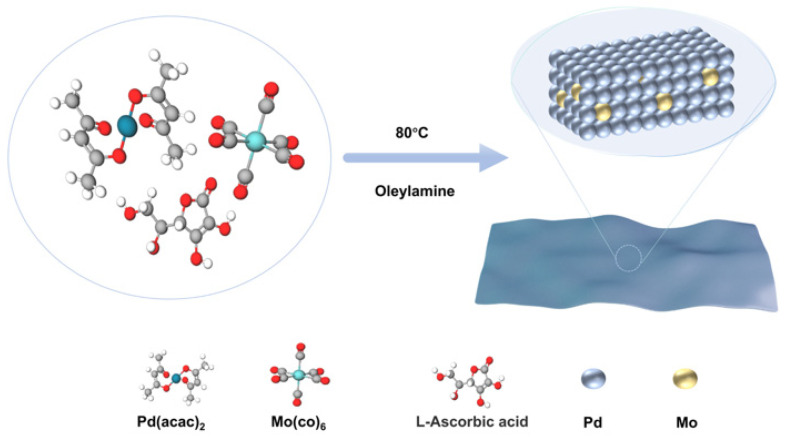
Schematic illustration of the facile synthesis process of PdMo bimetallene.

PdMo bimetallene is a highly curved, sub-nanometer-thick Pd–Mo alloy nanosheet. Owing to the modulation of its electronic structure by the alloying effect, strain effect arising from the curved geometry, and quantum size effect induced by sheet thinning, it has been demonstrated to be an efficient and stable electrocatalyst [20]. Figure 1 illustrates the facile synthesis process of PdMo bimetallene, with experimental details provided in the Experimental Section 4.2.

**Figure 2:**
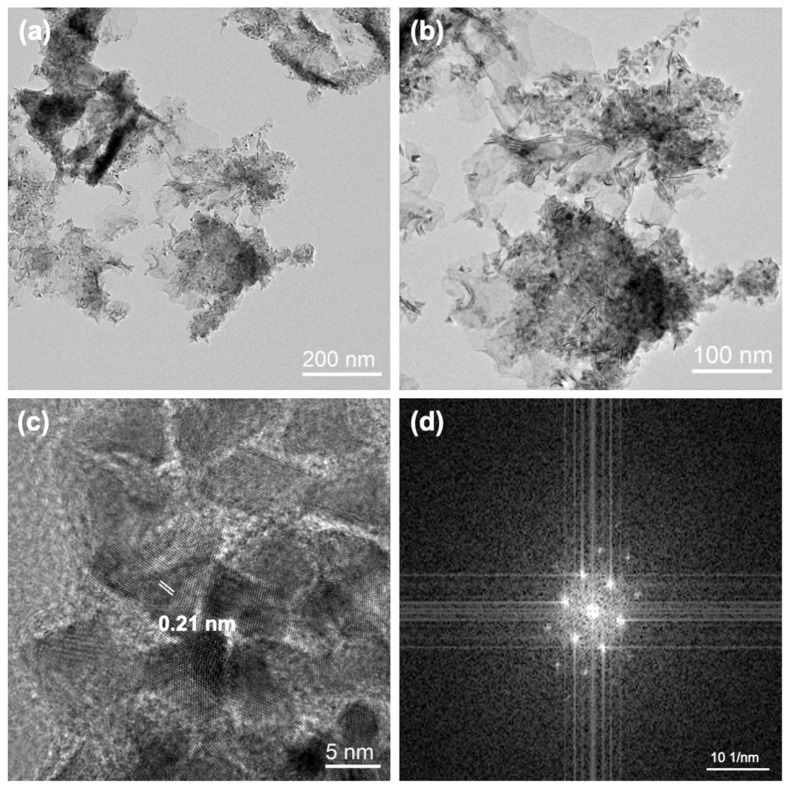
(**a**) Low-magnification TEM image of PdMo bimetallene (scale bar: 200 nm). (**b**) Magnified TEM image of PdMo bimetallene (scale bar: 100 nm). (**c**) HRTEM image of PdMo bimetallene showing a lattice spacing of 0.21 nm (scale bar: 5 nm). (**d**) FFT diffraction pattern derived from the HRTEM image, confirming the crystalline structure, preferred orientation, and periodic layered arrangement of the sample (scale bar: 10 1/nm).

The morphology of the as-synthesized product was first characterized by transmission electron microscopy (TEM). As shown in Figure 2a,b, the PdMo bimetallene exhibits a highly curved two-dimensional (2D) nanosheet structure with an average lateral size of several hundred nanometers, displaying typical bimetallene features [19]. The high-resolution transmission electron microscopy (HRTEM) image (Figure 2c) reveals continuous and highly distorted lattice fringes, with numerous grain boundaries and strained regions. As shown in Figure 2d, the fast Fourier transform (FFT) pattern obtained from the HRTEM image reveals the crystalline nature of PdMo bimetallene. Clear diffraction spots arranged with orthogonal symmetry are observed, confirming the long-range order of atomic arrangement and high crystallinity, indicating a preferred orientation along a specific zone axis. Multiple sets of equally spaced parallel diffraction streaks are clearly visible along the horizontal and vertical directions, corresponding to different families of crystal planes, suggesting the presence of periodic layered or superlattice structures within the material. Furthermore, slight broadening of the diffraction spots, along with the presence of diffuse scattering in the background, implies a certain degree of lattice strain, dislocations, or local structural disorder, while the overall crystalline order remains dominant. As shown in Figure 2c, quantitative analysis of the FFT pattern enabled precise determination of the lattice fringe spacing. The measured spacing is approximately 0.21 nm, which is notably smaller than the standard value for pure Pd (0.2246 nm, JCPDS No. 05-0681). The calculated lattice contraction ratio is approximately −6.50%. According to Hooke’s law (with the elastic modulus of Pd (111) being E = 121 GPa), this contraction corresponds to a significant compressive stress of approximately −7.87 GPa. This pronounced lattice contraction and compressive strain are attributed to the strong electronic interaction and atomic mismatch between Pd and Mo. These effects effectively modulate the d-band center of the Pd sites, optimizing the adsorption energy of DA intermediates and thereby contributing to the excellent electrocatalytic activity and DA sensing performance of the PdMo bimetallene-based sensor.

**Figure 3:**
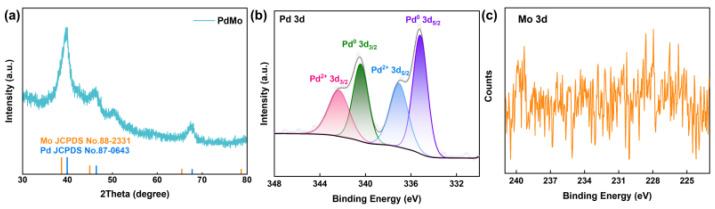
(**a**) XRD pattern of PdMo bimetallene, with standard diffraction lines of Mo (JCPDS No. 88-2331, Orange) and Pd (JCPDS No. 87-0643, Blue) shown as references. (**b**) High-resolution XPS spectrum of Pd 3d for PdMo bimetallene. (**c**) High-resolution XPS spectrum of Mo 3d for PdMo bimetallene.

The crystal structure and surface chemical state of the PdMo bimetallene sample were characterized by X-ray diffraction (XRD) and X-ray photoelectron spectroscopy (XPS), respectively (Figure 3). As shown in Figure 3a, the XRD pattern of PdMo bimetallene matches the standard diffraction lines of Pd (JCPDS No. 87-0643) and Mo (JCPDS No. 88-2331), confirming the successful preparation of the Pd–Mo bimetallic crystal. The broadened diffraction peaks indicate the presence of a nanoscale or ultrathin two-dimensional structure, which is favorable for exposing abundant active sites. Figure 3b displays the high-resolution Pd 3d XPS spectrum, which was deconvoluted into two pairs of peaks corresponding to the spin–orbit splitting of 3d_5/2_ and 3d_3/2_. The peaks located at 335.0 and 340.3 eV are attributed to metallic Pd^0^, with their peak areas dominating the spectrum, indicating that Pd predominantly exists in the metallic state, which ensures high electrical conductivity of the material and provides an efficient electron transport channel for electrochemical detection. The peaks at 336.2 and 341.5 eV correspond to oxidized Pd^2+^, suggesting partial surface oxidation; the Pd^2+^/Pd^0^ redox couple may further facilitate electron transfer at the electrode surface and enhance electrocatalytic activity. Compared with the standard binding energy of pure Pd^0^, the Pd^0^ 3d_5/2_ peak in the PdMo bimetallene exhibits a significant negative shift, directly confirming electron transfer from Mo to Pd, which is key evidence for the electronic synergistic effect between Pd and Mo. Regarding the Mo 3d XPS spectrum (Figure 3c), although the signal is relatively noisy due to the low Mo content, the characteristic spin–orbit splitting of Mo 3d (ΔE ≈ 3.1 eV) is still observed, and the broad peak shape suggests the presence of multiple Mo oxidation states (e.g., Mo^4+^, Mo^6+^), likely resulting from electronic interactions between Pd and Mo in the bimetallic structure. After electron transfer from Mo to Pd, the electron cloud density of Mo decreases, leading to an increased binding energy (positive shift); meanwhile, partial surface oxidation occurs, resulting in a multi-valence distribution. The multiple valence states of Mo (Mo^4+^/Mo^6+^) may act as an “electron shuttle” accelerating electron transfer between the electrode surface and DA molecules. These structural and electronic characteristics are expected to endow the PdMo bimetallene sample with excellent electrocatalytic activity for DA detection.

### 2.2. Electrochemical Performance Evaluation of the PdMo/GCE Sensor

**Figure 4:**
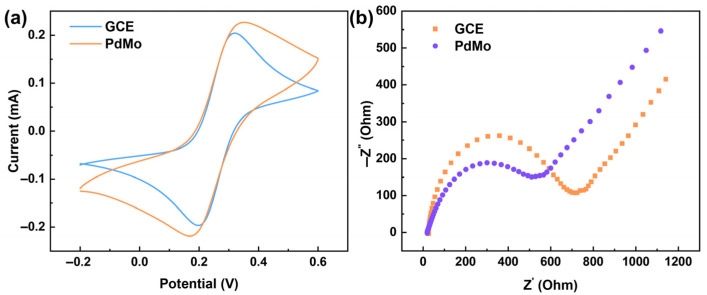
(**a**) CV curves of the working electrodes (GCE and PdMo bimetallene) after surface modification, recorded in a solution containing 1 mM K_3_[Fe(CN)_6_] and 100 mM KCl. (**b**) EIS spectra of the working electrodes (GCE and PdMo bimetallene) after surface modification, recorded in a solution containing 1 mM K_3_[Fe(CN)_6_] and 100 mM KCl.

To better understand and evaluate the improvement in the electrochemical response of the nanocatalytic material, cyclic voltammetry (CV) was performed for the PdMo/GCE and bare GCE electrodes in a solution containing 1 mM K_3_[Fe(CN)_6_] and 100 mM KCl. As shown in Figure 4a, the peak current of the GCE electrode modified with PdMo bimetallene is higher than that of the bare GCE. This enhancement is primarily attributed to the following factors: the ultrathin, highly curved two-dimensional bimetallene structure significantly increases the electrochemically active surface area (ECSA); the high metallic conductivity of the PdMo bimetallene facilitates accelerated interfacial electron transfer kinetics; and the synergistic effect between Pd and Mo optimizes the surface electronic structure. Collectively, these factors promote the redox reaction of the [Fe(CN)_6_]^3−/4−^ probe. Electrochemical impedance spectroscopy (EIS) was employed to characterize the electron transfer performance of the different electrodes, where the diameter of the semicircle in the high-frequency region typically represents the corresponding charge transfer resistance (Rct). Compared with the bare GCE electrode, the PdMo/GCE electrode exhibits a smaller Rct (601.2 Ω) (Figure 4b), indicating that PdMo bimetallene provides a fast electron transport pathway between the electrode and the electrolyte, along with a high electron transfer rate.

**Figure 5:**
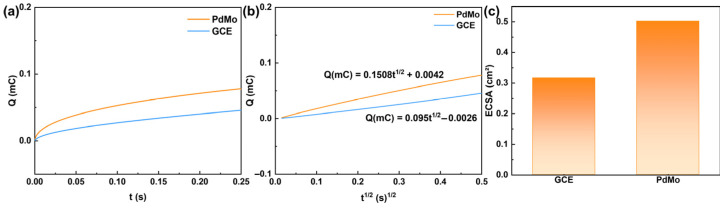
(**a**) Q–t plots and (**b**) Q–t^1/2^ plots of GCE and PdMo bimetallene. The experiments were performed in 100 mM KCl containing 1.0 mM K_3_[Fe(CN)_6_]. (**c**) Calculated ECSA of GCE and PdMo bimetallene.

The ECSA of the modified electrode was measured and calculated using chronocoulometry (CC). The chronocoulometric responses were recorded in a solution containing 1.0 mM K_3_[Fe(CN)_6_] and 100 mM KCl (Figure 5a). Figure 5b shows the relationship between Q and t^1/2^, and the ECSA was subsequently calculated based on the Anson equation [29]. The calculated ECSA values for the bare GCE and PdMo/GCE were 0.3165 cm^2^ and 0.5024 cm^2^, respectively (Figure 5c). After modification with PdMo bimetallene, the ECSA increased by approximately 58.7%, confirming that the ultrathin two-dimensional PdMo bimetallene effectively enlarges the ECSA and exposes a greater number of active sites. This finding is consistent with the enhanced redox peak currents observed in CV measurements, indicating the promising electrocatalytic potential of the PdMo/GCE electrode.

**Figure 6:**
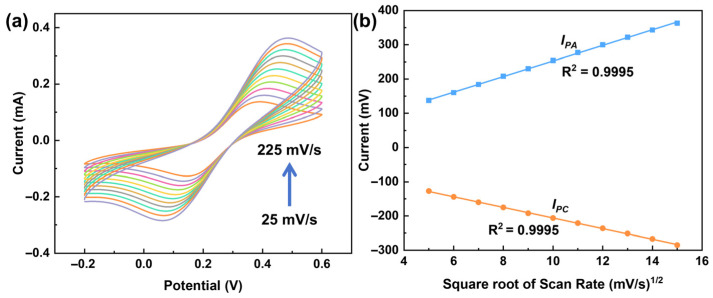
CV curves of PdMo bimetallene obtained in 1 mM K_3_[Fe(CN)_6_] and 100 mM KCl solution at different scan rates (25, 36, 47, 64, 81, 100, 121, 144, 169, 196, and 225 mV s^−1^) (**a**), and plots of current density versus the square root of scan rate (**b**).

To investigate the electrode reaction mechanism, the effect of scan rate was studied by CV in a solution containing 1 mM K_3_[Fe(CN)_6_] and 100 mM KCl. As shown in Figure 6, the peak currents of the redox peaks increase with increasing scan rate. The anodic peak current (*I_PA_*) and cathodic peak current (*I_PC_*) exhibit a good linear relationship with the square root of the scan rate, indicating that the redox process of [Fe(CN)_6_]^3−^ at the PdMo bimetallene surface is diffusion-controlled.

**Figure 7:**
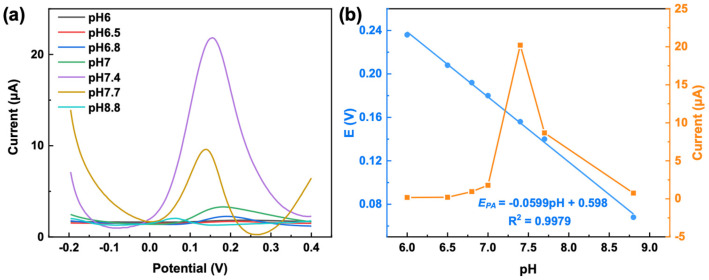
(**a**) DPV curves of 100 μM DA in 0.1 M PBS solution at various pH values (6–8.8). (**b**) Variations in *E_PA_* and *I_PA_* of DA with solution pH.

The pH of the electrolyte is a key factor regulating the protonation state of DA and the charge distribution on the electrode surface. In this study, the electrochemical response of DA over the pH range of 6.0–8.8 was investigated using DPV. As shown in Figure 7, with increasing pH, the oxidation peak potential (*E_PA_*) of DA shifted linearly negatively. The linear fitting equation was $E_{PA}\;=\;-0.0599pH\;+\;0.598$ ($R^{2}\;=\;0.9979$). According to the Nernst equation, the calculated m/n value for DA oxidation was 1.01, indicating that the oxidation of DA on this electrode proceeds via a two-electron, two-proton equivalent transfer process, which is fully consistent with the classical oxidation mechanism of DA. In the pH range of 6.0–7.4, the peak current (*I_PA_*) continuously increased with rising pH. When the pH exceeded 7.4, *I_PA_* significantly decreased with further increase in pH. Thus, pH 7.4 represents the optimal reaction condition for DA oxidation, at which the electrochemical sensitivity of the electrode toward DA reaches its maximum. This value is in good agreement with physiological pH, confirming that this pH condition is suitable for the practical detection scenarios of DA sensors.

### 2.3. PdMo/GCE Sensor for the Electrochemical Detection of Dopamine

**Figure 8:**
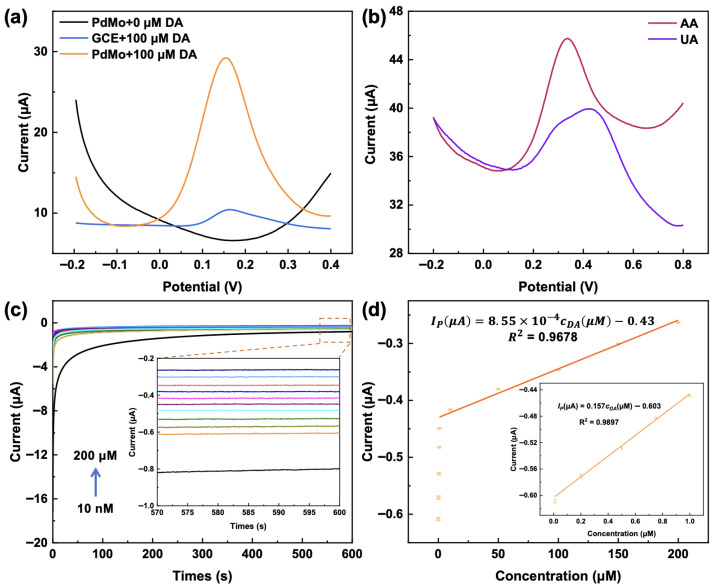
(**a**) DPV responses of the sensors before and after surface modification with PdMo bimetallene, measured after incubation with 100 µM DA in 0.1 M PBS (pH 7.4). (**b**) DPV responses of the PdMo/GCE sensor to 1.0 mM AA in 0.1 M PBS (pH 7.4) solution and 1.0 mM UA in 0.1 M PBS (pH 7.4) solution. (**c**) i–t responses of the PdMo bimetallene-based sensor to DA concentrations ranging from 10 nM to 200 µM. (**d**) Linear relationship between the response current and DA concentration over the range of 10 nM to 200 µM.

In this study, PdMo bimetallene nanomaterial was loaded onto a GCE to construct a high-performance bioelectrochemical sensor for DA. On the PdMo bimetallene surface, DA undergoes a reversible oxidation reaction to form dopamine quinone (DOQ), with the release of two electrons. To evaluate the catalytic performance of the modified electrode toward DA, Differential pulse voltammetry (DPV) was performed in a 0.1 M phosphate-buffered saline (PBS, pH 7.4) containing 100 µM DA. As shown in Figure 8a, compared with the bare GCE electrode, the PdMo/GCE electrode exhibits a higher peak current and a lower peak potential (0.156 V), indicating that a greater amount of DA undergoes the electrochemical reaction at a specific potential, and that the catalyst facilitates the reaction with lower energy cost. This suggests that modification with PdMo bimetallene effectively enhances the electrocatalytic activity of the electrode. As shown in Figure 8b, the oxidation peak potentials of AA and UA were +0.335 V and +0.436 V, and the oxidation onset potentials were +0.1 V and +0.2 V, respectively. The specific electrocatalytic effect of the PdMo bimetallene significantly reduced the oxidation overpotential of DA, further increasing the difference in oxidation potentials between DA and AA, UA.

As confirmed by the chronoamperometric (i–t) curve in Figure 8c, the PdMo bimetallene exhibits high sensitivity to DA at an applied potential of +0.05 V. The electrode shows a rapid and stable current response, with the steady-state current monotonically increasing with rising DA concentration, and functions effectively across the DA concentration range of 10 nM to 200 µM, indicating a clear positive correlation between the response current and the analyte concentration. The calibration curve presents two consecutive linear ranges for quantitative detection. In the low DA concentration range of 0.01–1 µM, the regression equation is expressed as $Ip\left(\mu A\right)=0.157c_{DA}\left(\mu M\right)-0.603$ (R^2^ = 0.9897). In the high DA concentration range of 1–200 µM, the regression equation is expressed as $Ip\left(\mu A\right)=8.55\times 10^{-4}c_{DA}\left(\mu M\right)-0.43$ (R^2^ = 0.9678) (Figure 8d). The sensor exhibits a high sensitivity of 80 µA µM^−1^ cm^−2^ in the low concentration range. Based on a signal-to-noise ratio (S/N = 3), the limit of detection (LOD) is calculated to be 0.14 µM.

The PdMo bimetallene sensor was compared with other recently reported DA sensors, and the results are summarized in Table 1. Compared with other advanced sensors based on two-dimensional materials or noble metal nanoparticles, the PdMo/GCE exhibits a wider linear range (0.01–200 µM) and excellent sensitivity (80 µA·µM^−1^·cm^−2^). Regarding the detection limit, although some reported sensors (e.g., PdNPs/4AP N-GQDs and Ni-Pt@ZIF-8) achieve lower detection limits under ideal conditions (21 pM and 1 nM, respectively), their linear ranges are narrow, limiting their practical applicability across wide concentration ranges. In contrast, the PdMo/GCE provides a linear dynamic range spanning four orders of magnitude while maintaining a favorable detection limit (0.14 µM), making it more suitable for monitoring fluctuations in DA concentrations in real biological samples. Furthermore, benefiting from the unique two-dimensional structure of the bimetallene, the synergistic electronic effect between Pd and Mo, and the abundant active sites, the PdMo/GCE exhibits significantly higher sensitivity than advanced materials such as TiO_2_/MXene/rGO (21.893 µA·µM^−1^). The above comparison highlights the distinctive advantages of the bimetallene concept in biosensor design.

### 2.4. Selectivity, Reproducibility, and Repeatability of the PdMo/GCE Sensor and Its Detection in Biological Samples

**Figure 9:**
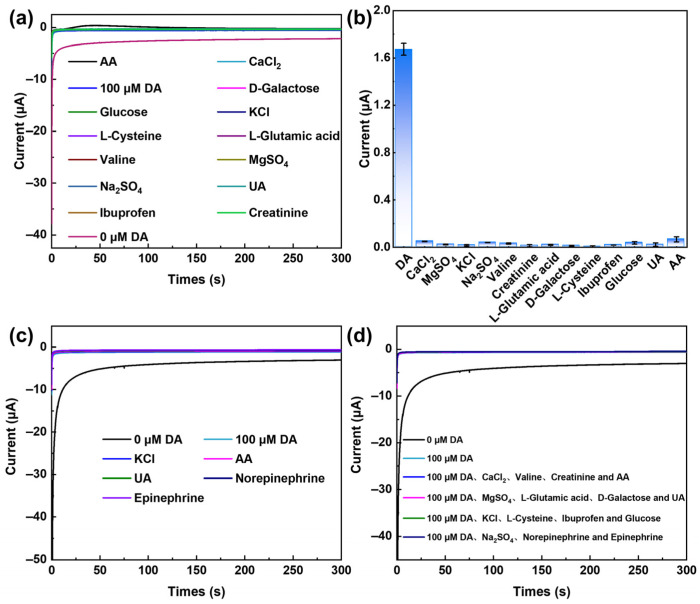
(**a**) Anti-interference capability of the PdMo bimetallene-based sensor for the detection of 100 μM DA, and (**b**) the corresponding bar chart statistics. (The concentration of each interfering species is 1.0 mM. Interfering species: CaCl_2_, MgSO_4_, KCl, Na_2_SO_4_, Valine, Creatinine, L-Glutamic acid, D-Galactose, L-Cysteine, Ibuprofen, Glucose, UA, AA). (**c**) Anti-interference capability of the PdMo bimetallene-based sensor in the presence of ultra-high concentrations of interferents. (Interferent concentration: 10 mM. Interfering species: KCl, AA, UA, Norepinephrine, and Epinephrine). (**d**) Anti-interference capability of the PdMo bimetallene-based sensor in mixed interferent solutions. (All mixed solutions contain 100 μM DA and various interferents at 10 mM. Mixture 1: DA, CaCl_2_, Valine, Creatinine, and AA; Mixture 2: DA, MgSO_4_, L-Glutamic acid, D-Galactose, and UA; Mixture 3: DA, KCl, L-Cysteine, Ibuprofen, and Glucose; Mixture 4: DA, Na_2_SO_4_, Norepinephrine, and Epinephrine).

DA, as a critical neurotransmitter, is often subject to interference from coexisting species such as AA and UA during selective detection in biological samples including urine and brain tissue [14]. Therefore, investigation of the selectivity of the PdMo/GCE sensor is of particular importance. i–t analysis was conducted in the presence of a tenfold excess of common biological substances, including CaCl_2_, MgSO_4_, KCl, Na_2_SO_4_, Valine, Creatinine, L-Glutamic acid, D-Galactose, L-Cysteine, Ibuprofen, Glucose, UA, and AA. As shown in Figure 9a,b, no significant amperometric response was observed upon the addition of these interfering species, indicating satisfactory specificity of the sensor. In general, other methods for DA detection based on oxidation current are often susceptible to interference from easily oxidizable substances such as AA, UA, and cysteine at low potentials [35,36]; in contrast, the present material exhibits distinct advantages. Considering that in real biological samples (e.g., serum), the concentrations of interfering species are typically much higher than that of DA, to further validate the anti-interference performance of the sensor in practical application scenarios, we increased the concentrations of the main interferents (KCl, AA, UA, Norepinephrine, and Epinephrine) to 100 times the DA concentration (100 µM). As shown in Figure 9c, even in the presence of these high-concentration interferents, no significant change in the amperometric response of the DA oxidation peak current was observed, fully confirming that the PdMo/GCE sensor possesses excellent high-concentration anti-interference capability, thereby meeting the requirements for accurate detection of DA in real biological samples.

Furthermore, the steady-state current response of the sensor upon the addition of several interfering species was investigated in 0.1 M PBS (pH 7.4) containing 100 µM DA (Figure 9d). Four groups of mixed interference solutions containing 100 µM DA were prepared, each supplemented with high concentrations (10 mM) of inorganic ions, amino acids, sugars, common drugs, and structurally similar neurotransmitters. The test results indicate that regardless of the combination of interfering species, the sensor exhibits acceptable selectivity, with a maximum change in the DA current response of only 8.64%. These findings confirm that the constructed sensor possesses excellent selective recognition capability for DA even in complex systems with multiple coexisting interferents.

**Figure 10:**
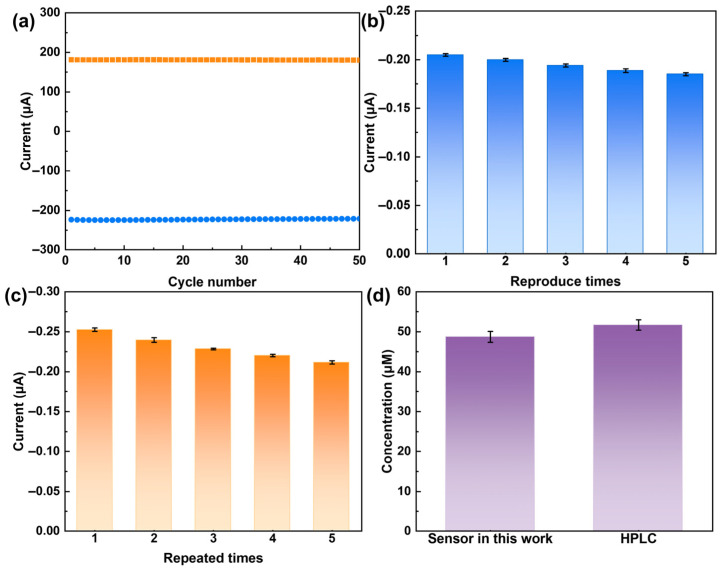
(**a**) Anti-fouling performance of the PdMo bimetallene-based sensor for DA detection. (**b**) Reproducibility of the PdMo bimetallene-based sensor in 0.1 M PBS (pH 7.4) containing 100 µM DA (*n* = 5). (**c**) Repeatability of the PdMo bimetallene-based sensor in 0.1 M PBS (pH 7.4) containing 100 µM DA (*n* = 5). (**d**) Comparison of DA detection between the constructed PdMo bimetallene sensor and HPLC.

To evaluate the anti-fouling capability of the PdMo/GCE sensor, 50 consecutive CV scans were performed. As shown in Figure 10a, both the anodic and cathodic peak currents of DA slightly decreased with increasing scan number. After the 50th scan, the anodic peak current decreased by approximately 0.607% relative to the first scan, while the cathodic peak current decreased by approximately 1.074%. This result indicates that the PdMo/GCE sensor exhibits good electrochemical stability and anti-fouling capability during continuous scanning. This minor current decay is primarily attributed to the physical adsorption of a small amount of DA oxidation products on the electrode surface. Notably, the atomically thin layered structure and electron-rich surface of PdMo bimetallene can effectively reduce the accumulation of strongly adsorbed species. Moreover, its curved morphology may facilitate local mass transport, further mitigating the buildup of strongly adsorbed oxidation products. These structural features collectively endow the PdMo/GCE sensor with excellent anti-fouling performance, providing an important foundation for its application in real clinical detection.

Meanwhile, the repeatability and reproducibility of the PdMo/GCE sensor were also investigated. As shown in Figure 10b, after five consecutive measurements using the same modified working electrode, the amperometric response remained above 90.3% of its initial value, with a relative standard deviation (RSD) of 4.03%, indicating good reproducibility of the sensor. As shown in Figure 10c, the RSD of the amperometric response for five batches of independently prepared working electrodes in 0.1 M PBS (pH 7.4) containing DA (100 µM) was 6.86%, demonstrating satisfactory repeatability.

To evaluate the practical applicability of the sensor in biologically relevant environments, fetal bovine serum (FBS) diluted 100-fold was used as the actual sample matrix, and the detection performance for DA was investigated using the standard addition method. i–t tests were performed after spiking the samples with DA solutions at different concentrations (0.6 µM, 20 µM, and 170 µM). As shown in Table 2, the recovery rates of DA in the FBS samples ranged from 102.03% to 112.53%, with RSD values between 0.23% and 1.50%. To further validate the performance of the constructed sensor, the detection results for DA obtained using the PdMo/GCE sensor were compared with those from the HPLC method. As shown in Figure 10d, no significant difference was observed between the results obtained with the constructed sensor and those from the HPLC method, confirming the high accuracy of the sensor for real-time monitoring of DA concentrations in the brain and its significant potential for commercial applications.

## 3. Discussion

In this study, a novel electrochemical sensor based on PdMo bimetallene was successfully developed for the selective and sensitive detection of DA. A highly curved, atomically thin bimetallene structure was synthesized via a facile wet-chemical method and systematically characterized. The as-prepared PdMo bimetallene-modified electrode exhibits excellent electrocatalytic activity toward DA oxidation, which is attributed to the synergistic electronic effect between Pd and Mo, the high density of active sites arising from the two-dimensional structure, and the favorable lattice strain. Among these features, the atomically thin layered structure and curved morphology provide a high density of edge/defect active sites, significantly increasing the reaction interface. Meanwhile, the compressive strain arising from lattice mismatch further optimizes the d-band center of the Pd sites, thereby modulating the adsorption strength of DA intermediates. Collectively, these factors promote the enhancement of catalytic efficiency. The sensor demonstrates a wide linear range (0.01–200 µM), a low detection limit (0.14 µM), an exceptionally high sensitivity (80 µA·µM^−1^·cm^−2^), and outstanding selectivity, enabling effective discrimination of DA from common interfering species such as AA and UA. Its good reproducibility and repeatability further highlight its potential for practical applications. This study not only establishes PdMo bimetallene as a high-performance electrochemically active material for biosensing but also opens new avenues for the application of bimetallene nanostructures in the detection of other neurotransmitters and biologically relevant molecules. Future work will focus on integrating this sensor into flexible wearable platforms to enable continuous monitoring of DA in real biological fluids.

## 4. Materials and Methods

### 4.1. Materials and Reagents

Palladium diacetylacetonate (Pd(acac)_2_, 99%), Molybdenum hexacarbonyl (Mo(CO)_6_, ≥99.9%), AA, oleylamine, cyclohexane, Nitric acid (HNO_3_, 69%), anhydrous ethanol, isopropanol (99.9%), Potassium ferricyanide, CaCl_2_, MgSO_4_, KCl, Na_2_SO_4_, Valine, Creatinine, L-Glutamic acid, D-Galactose, L-Cysteine, Ibuprofen, Glucose, UA, AA, Epinephrine, Norepinephrine and DA were purchased from Sigma-Aldrich (St. Louis, MO, USA). All chemicals were of analytical grade and used without further purification. Deionized water (UP, resistivity 18.2 MΩ cm^−1^, Merck Millipore, Burlington, MA, USA) was used throughout the experiments. PBS (0.1 M, pH 7.4) was employed as the supporting electrolyte.

### 4.2. Synthesis of PdMo Bimetallene

In a typical synthesis of PdMo bimetallene, 10 mg of Pd(acac)_2_, 30 mg of AA, 4 mg of Mo(CO)_6_, and 5 mL of oleylamine were mixed in a 20 mL glass vial. The mixture was purged with nitrogen (N_2_) for 1 h before being sealed and then sonicated until a homogeneous solution was formed. The sealed vial was transferred to an oil bath and heated at 80 °C for 12 h [15]. After cooling to room temperature, the black colloidal product (PdMo bimetallene) was collected by centrifugation (8000 rpm, 10 min) and further washed with a mixture of cyclohexane and anhydrous ethanol (*v*:*v* = 1:2) to remove excess oleylamine. The collected product was then dried in an oven at 30 °C for subsequent use.

### 4.3. Material Characterization

The morphology and crystal structure of the as-synthesized PdMo bimetallene were observed using TEM (JEOL JEM-2100F, FEI Company of the United States, Hillsboro, OR, USA, operating at an accelerating voltage of 200 kV) and HRTEM (JEOL, Tokyo, Japan). Surface chemical composition and electronic states were analyzed by XPS (Thermo Fisher Scientific K-Alpha, Thermo Fisher Scientific, Waltham, MA, USA, with an Al Kα X-ray source). The crystalline phase was identified using XRD (Bruker D8 Advance, Bruker AXS GmbH, Karlsruhe, Germany, with Cu Kα radiation).

### 4.4. Electrode Modification and Electrochemical Measurements

The GCE(diameter 5 mm) was polished to a mirror finish using Al_2_O_3_ slurry (particle size 0.02–0.05 μm), followed by sequential ultrasonic cleaning in 50% nitric acid (HNO_3_) solution, anhydrous ethanol, and UP for 5 min each. After drying, the electrode was set aside for use. All electrochemical experiments were performed on a CHI 760E electrochemical workstation (CH Instruments, Austin, TX, USA) using a standard three-electrode system at room temperature. A saturated Ag/AgCl electrode was used as the reference electrode, a Pt wire as the counter electrode, and the PdMo/GCE as the working electrode.

Briefly, a catalyst ink was prepared by mixing 2 mg of PdMo bimetallene with 1 mL of isopropanol. The mixture was then ultrasonicated for 30 min (ultrasonic power: 100 W, water bath temperature: 25 °C) to form a homogeneous catalyst slurry. Finally, 10 μL of the catalyst ink was deposited onto a clean GCE and dried in an oven at 30 °C until the solvent completely evaporated. The as-coated electrode was used as the working electrode for subsequent electrochemical measurements without additional rinsing.

CV was employed to characterize the conductivity of the modified and bare electrodes in a solution containing 1 mM K_3_[Fe(CN)_6_] and 100 mM KCl, with a potential range of −0.2 to 0.6 V at a scan rate of 50 mV·s^−1^. The anti-fouling capability of the sensor was similarly characterized by performing 50 consecutive CV scans in a 0.1 M PBS solution (pH 7.4) containing 100 μM DA, with a potential range from −0.2 to 0.6 V and a scan rate of 50 mV·s^−1^.

Qualitative and quantitative analyses of the peak potential and peak intensity of the samples were performed using DPV in a 0.1 M PBS solution (pH 7.4) containing 100 μM DA, as well as in solutions with different pH values (6–8.8). The measurements were carried out with a potential range from −0.2 to 0.4 V, a pulse width of 0.06 s, a pulse period of 0.5 s, and an amplitude of 50 mV.

Qualitative and quantitative analyses of the peak potential and peak intensity of the samples were performed using DPV in 0.1 M PBS solutions (pH 7.4) containing 1.0 mM AA and 1.0 mM UA, respectively. The measurements were conducted with a potential range from −0.2 to 0.8 V, a pulse width of 0.06 s, a pulse period of 0.5 s, and an amplitude of 50 mV.

The ECSA of the modified electrode was measured and calculated using CC in a solution containing 1.0 mM [Fe(CN)_6_]^3−/4−^ and 100 mM KCl. The measurement parameters were as follows: initial potential 0.6 V, step potential −0.1 V, and pulse width 0.25 s. The relationship between the charge (Q) and the square root of time (t^1/2^) was recorded and analyzed according to the Anson equation:

$$Q\left(t\right)=\frac{2nFACD^{1/2}t^{1/2}}{\pi^{1/2}}+Q_{dl}+Q_{ads}$$

where *n* is the number of electrons transferred (for the [Fe(CN)_6_]^3−/4−^ redox couple, *n* = 1), F is the Faraday constant (96,485 C·mol^−1^), C is the concentration of K_3_[Fe(CN)_6_] (1 × 10^−6^ mol·cm^−3^), and D is the diffusion coefficient of [Fe(CN)_6_]^3^ (7.6 × 10^−6^ cm^2^ s^−1^). The ECSA (A) was calculated from the slope of the Q–t^1/2^ plot and used to normalize the sensor sensitivity, allowing a more accurate reflection of the intrinsic catalytic performance of the material.

The amperometric response of the sensor to DA was characterized using i–t in 0.1 M PBS (pH 7.4) with varying concentrations of DA. A calibration curve was constructed at an applied potential of +0.05 V, with a sampling interval of 0.1 s and an establishment time of 0 s. Electrochemical impedance spectroscopy (EIS) was performed in a solution containing 1 mM K_3_[Fe(CN)_6_] and 100 mM KCl at open-circuit potential, with a frequency range of 10^−1^ to 10^5^ Hz and an amplitude of 5 mV.

Reproducibility was assessed by evaluating the amperometric response of five independently prepared PdMo/GCE electrodes to 100 µM DA using i–t. After the background current stabilized at an applied potential of +0.05 V, 100 µM DA was added to the stirred 0.1 M PBS (pH 7.4), and the steady-state current response was recorded. The RSD of the response currents from the five electrodes was calculated to evaluate the reproducibility of the electrode fabrication process.

Repeatability was evaluated by measuring the stability of a single PdMo/GCE electrode during five consecutive measurements of 100 µM DA using i–t under the same experimental conditions. After each measurement, the electrode was rinsed with PBS buffer and allowed to rest at room temperature for 5 min to restore its initial state. The RSD of the response currents from the five measurements was calculated to assess the repeatability of the electrode measurements.

Interference studies were conducted by sequentially adding DA (100 µM) and interfering species (CaCl_2_, MgSO_4_, KCl, Na_2_SO_4_, Valine, Creatinine, L-Glutamic acid, D-Galactose, L-Cysteine, Ibuprofen, Glucose, UA, and AA, 1 mM) to 0.1 M PBS (pH 7.4), with the current response recorded using i–t. To evaluate the selectivity of the PdMo/GCE sensor under extremely high concentrations of interfering species, experiments were conducted by sequentially adding DA (100 µM) and interfering substances (KCl, AA, UA, Norepinephrine, Epinephrine; 10 mM) to 0.1 M PBS (pH 7.4). To further assess the selectivity of the PdMo/GCE sensor for DA in the presence of multiple coexisting interferents, four complex mixtures were prepared (Mixture 1: DA, CaCl_2_, Valine, Creatinine and AA; Mixture 2: DA, MgSO_4_, L-Glutamic acid, D-Galactose and UA; Mixture 3: DA, KCl, L-Cysteine, Ibuprofen and Glucose; Mixture 4: DA, Na_2_SO_4_, Norepinephrine and Epinephrine), each containing 100 µM DA and various coexisting species at a concentration of 10 mM. All experiments were performed using i–t to record the current responses. All measurements were carried out at room temperature.

The recovery rates of DA were determined by i–t tests in 100-fold diluted FBS solutions containing different DA concentrations (0.6 µM, 20 µM, and 170 µM).

## Figures and Tables

**Table 1:** Comparison of analytical performance of various electrochemical sensors for DA detection.

Electrode Material	Method	Linear Range (µM)	LOD (µM)	Sensitivity	Reference
PdMo bimetallene	i–t	0.01–200	0.14	80 µA·µM^−1^·cm^−2^	This work
Ni−Pt@ZIF-8	DPV	0.001–10	0.001	—	[30]
PdNPs/4AP N-GQDs	DPV	0.00025–0.01	0.000021	—	[31]
TiO_2_/MXene/rGO	DPV	0.01–2.4/2.4–50	0.0051	21.893 µA·µM^−1^	[32]
Au@MoS_2_	DPV	0.8–10	0.0789	—	[33]
Ti_3_C_2_T_X_@TiO_2_ NSs	DPV	40–300	0.19	—	[34]

**Table 2:** Detection results of DA in FBS samples using the PdMo/GCE sensor.

Samples	Method	Spiked (µM)	Determined (µM)	Recovery (%)	RSD (*n* = 3) (%)
FBS	i–t	0.6	0.65	108.93	0.23
		20	0.001	112.53	1.50
		170	173.45	102.03	0.27

## Data Availability

The original contributions presented in this study are included in the article. Further inquiries can be directed to the corresponding author.
